# PRN Medicines Management for Psychotropic Medicines in Long-Term Care Settings: A Systematic Review

**DOI:** 10.3390/pharmacy7040157

**Published:** 2019-11-25

**Authors:** Mojtaba Vaismoradi, Flores Vizcaya Moreno, Hege Sletvold, Sue Jordan

**Affiliations:** 1 Faculty of Nursing and Health Sciences, Nord University, 8049 Bodø, Norway; hege.sletvold@nord.no; 2 Faculty of Health Sciences, University of Alicante, E-03080 Alicante, Spain; flores.vizcaya@ua.es; 3 College of Human and Health Sciences, Swansea University, Swansea SA2 8PP, UK; s.e.jordan@swansea.ac.uk

**Keywords:** *pro re nata*, PRN, medicines management, nurse, patient safety, psychotropic medications, long-term care setting

## Abstract

Many medications are prescribed and administered PRN (*pro re nata*, as needed). However, there are few integrative reviews to inform PRN psychotropic medication use in long-term care facilities and nursing or care homes. Accordingly, this integrative systematic review aimed to improve our understanding of PRN medicines management with a focus on psychotropic medications (antipsychotics, sedatives, anxiolytics, and hypnotics) in long-term care settings. Keywords relating to PRN in English, Norwegian, and Spanish were used, and articles published between 2009 and 2019 were retrieved. Based on the inclusion criteria, eight articles were used for data analysis and synthesis. This review offers a description of PRN prescription and administration of psychotropic medications in long-term care. Variations were observed in the management of PRN psychotropic medications based on residents’ underlying health conditions and needs, duration of use, and changes between medications and doses. Neither the reasons for PRN prescription and administration nor the steps taken to identify and manage any associated adverse reactions or adverse drug events were reported. Further initiatives are needed to improve PRN medicines management to explore factors that affect PRN prescription and administration and to develop appropriate PRN guidelines to prevent harm and improve the safety of people living in long-term care facilities.

## 1. Introduction

PRN stands for “*pro re nata*,” and indicates authorising nurses to administer medications according to patients’ requests and nurses’ discretion. PRN is categorized as unscheduled medication administration either alone or in addition to routine/regular prescriptions [1]. It is frequently used for medications prescribed by physicians and administered based on nurses’ judgements of patients’ needs [2,3,4,5,6,7], but PRN medications are sometimes self-administered or given by family or informal caregivers [2]. PRN is commonly used to prescribe medications required in particular conditions, such as a complement to regularly scheduled medications [8]. It empowers nurses and patients and gives them more flexibility for relieving unpleasant physical and mental symptoms [2,9]. Moreover, active involvement in decision-making regarding patient care, including medicines management, can be considered to increase professional autonomy, with the potential to increase the sense of self-worth and accomplishment in practice [10]. However, PRN medication decision-making for nurses is complex, and is influenced by pharmacotherapeutic competencies and skills, patient and family involvement, and organizational routines [11,12]. Decision-making is a key skill for many healthcare professionals and is important to ensure patient safety [13]. 

PRN prescription and administration is commonly used for psychotropic and psycholeptics medications, including antipsychotics, neuroleptics, anxiolytics, sedatives, and hypnotics [6,8,9,14,15,16,17,18], analgesics [6,12,14,19,20,21], gastro-intestinal preparations [12,14], and other physical and psychological symptoms [2]. Benefits of PRN administration to patients are noted [2,9], but the use of PRN psychotropic medications has been linked to an increased risk of falls [22,23,24], infection, or dehydration [24]. PRN medication is associated with dementia diagnosis, older age, polypharmacy, and a longer length of stay in nursing homes [8,25]. Additionally, higher rates of PRN administration have been associated with high dependency in daily living activities [25], individual care homes [12,26], polypharmacy, increased risk of dependence, overdose, and overuse of medications [4,8,24,27], but more exploration is needed [25,26]. PRN can be linked to administration without the patient’s consent or full disclosure of relevant information about PRN medications, particularly to patients suffering from cognitive impairments [3,28]. While potentially inappropriate use of PRN medications [24] has been reported, one study of PRN medication administration based on medication charts in residential aged care services has shown that rates of administration of PRN medications are lower than anticipated [12]. However, this might have been attributed to a lack of documentation, poor concordance between written notes and verbal reports [16,29], and the use of self-report and phone interviews to assess PRN medication use [30]. Since then, the use of electronic health records in hospitals has increased the accuracy of data collection on the quantity of PRN medication, its prescription, and its administration [16].

PRN medicine use in long-term care settings has long been considered integral to medicines management systems. However, there are few systematic reviews of PRN medicines management in long-term care settings, and those available provide an overall description of PRN only in nursing homes and without a focus on psychotropics medicines [25], the class of medications most commonly administered to residents as PRN. We did not identify reviews of PRN psychotropic medication use in long-term care settings. Therefore, the present systematic review aims to address this gap, and improve understanding of PRN medicines management.

## 2. Materials and Methods 

### 2.1. Design of the Study

This integrated systematic review considered both qualitative and quantitative papers [31,32,33] on PRN medicines management with a focus on psychotropic medications in long-term care settings. The integrative approach provided the opportunity to incorporate individuals’ understanding gained from qualitative studies with statistical data and present a more comprehensive image of the study phenomenon [34].

### 2.2. Search Strategy and Data Collection

The authors’ experiences in the field of medicines management and a pilot search in international general and specialized databases helped to identify appropriate keywords. Boolean search methods were used to identify articles on PRN medicines management of psychotropic medications in long-term care settings using the following terms: “PRN” (*pro re nata*) OR “as needed” OR “as required” AND psychotropic/s OR antipsychotic OR neuroleptic OR anxiolytics OR sedatives OR psycholeptic/s OR anxiolytics OR hypnotics OR risperidone OR haloperidol OR lorazepam OR temazepam OR phenothiazines AND “long-term” OR home OR “home health nursing” OR “home nursing.” The above-mentioned keywords were translated to Norwegian and Spanish, and a similar systematic search in Nordic and Spanish scientific databases was conducted. Online databases (PubMed [including Medline], Scopus, Cinahl, Cochrane library, Norart, SweMed, IBECS, Cuiden, and Medes) were searched to retrieved articles published from 2009 to 2019 in scientific journals. Inclusion criteria were a focus on PRN prescription and administration for psychotropic medications in long-term care settings, and publication in peer-reviewed journals. 

### 2.3. Systematic Review and Quality Appraisal 

The search process was performed independently by the authors (MV, MFV, and HS), but they held online discussions to share their results and make decisions on the further steps of the review. The search process identified 1594 articles (Table 1). 

A pre-piloted data extraction table was used to extract the studies’ core details in terms of general characteristics of studies, methods, sample, settings, and relevant results. Title readings, deletion of duplicates, and selection of relevant studies to the review topic led to 54 articles that were shared between the authors to ensure their suitability based on mutual agreements for inclusion in the next review step. In addition, a manual search was conducted in the more well known journals in the field of pharmacy, caring science, and medicines management, and no more studies were identified. Abstracts were read by each author. Those with a possible focus on PRN medicines management and the use of psychotropic medications in long-term care settings were selected (*n* = 8). The full texts of these 8 articles were obtained from Norwegian and UK libraries. They were assessed for relevance and focus on the study topic, and all 8 were retained. To improve the search coverage, gray literature and cross-references from bibliographies were reviewed for additional studies, and a manual search was performed in the reference lists of the 8 studies, and no more articles were identified. Therefore, the selected articles (*n* = 8) were appraised in terms of methodological transparency and soundness through the Enhancing the QUAlity and Transparency of Health Research (EQUATOR) tools. Accordingly, appraisal tools appropriate to each study’s methodology (including STROBE [for cross-sectional, observational, and cohort studies] and COREQ [for qualitative studies]) were used to evaluate the eligibility of each article for inclusion in data analysis in terms of research framework, findings, and conclusion [35]. Since items of the quality appraisal tools for making a decision for an article’s inclusion did not have an equal weight, no scoring system was used. Therefore, discussions on the importance and quality of each article were held between researchers to reach an agreement on the selection of articles for data analysis and synthesis. The quality appraisal led to no exclusion of articles. Table 2 summarises the characteristics of the selected articles for inclusion in data analysis and synthesis. 

**Figure 1:**
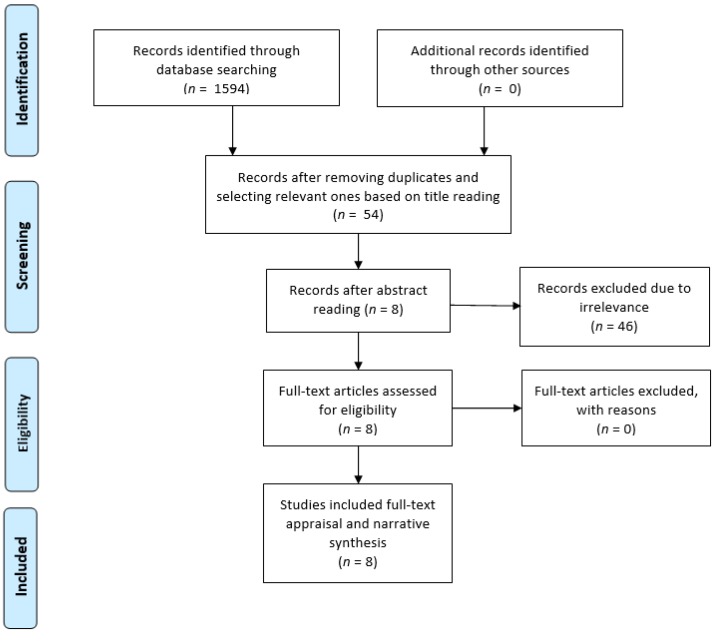
The study flow diagram based on the PRISMA statement.

The following schematic diagram illustrates the inclusion criteria of reporting items for systematic review and is based on the meta-analysis (PRISMA) statement [36] (Figure 1).

## 3. Results

### 3.1. Description of the Selected Studies

The selected studies comprised three studies from Australia [37,43,44], one from the USA [38], one from Norway [39], one from Canada [40], and two from Germany [41,42]. They were all published in English except one that was in Norwegian [39]. Most studies used quantitative methods, including cross-sectional surveys [37,39,41,42,44], a secondary analysis of a prospective cohort [40], and a prospective longitudinal intervention study [43]. There was one qualitative study of interviews and documentations [38].

### 3.2. Prevalence and Type of PRN Prescription and Administration

Variations were found in the prevalence of PRN prescription and administration, indicating differences in routines for PRN medicines management. In addition, variations in reporting PRN medications in terms of percentages and means hindered comparison of the studies’ findings and derivation of a single overall figure. Prevalence ranged from 1.1% of prescriptions [39] to 35.9% of prescriptions [42], depending on residents’ underlying conditions. Additionally, a few studies provided data on the actual administration of prescribed PRN medications, rather than prescriptions. Medicines prescribed and administered PRN to residents in long-term care settings included anxiolytics, hypnotics, and antipsychotics under different names and brands (Table 3). 

### 3.3. Factors Affecting PRN Medicines Management

PRN prescription and administration was associated with residents’ behaviours and needs, including seeking attention and disturbing others during the night [38,40]. Decisions to administer PRN medications were informed by residents’ verbal requests, nonverbal cues, interpretation of residents’ behaviours, and the settings’ characteristics in terms of staffing pattern, storage and documentation of medications, and circumstances for use, discontinuation, and reporting [38]. Education, experience, and interdisciplinary interventions improved antipsychotic and benzodiazepine medicines management in terms of reduction of overall PRN prescription and administration [43]. While demographic characteristics of residents in terms of age and gender were not associated with PRN prescription and administration, higher numbers of PRN medications were associated with increased use of long-term medications (7.4 ± 3.5) and a longer duration of stay (4.8 ± 4.3 years). Dementia diagnosis and older age were associated with more PRN medication prescription and administration [41,42]. Additionally, a longer stay, above the median of 2.1 years, was associated with 2.38 more PRN medications [41]. 

## 4. Discussion

In this review, the prevalence of PRN prescribing in long-term care settings is varied. Variations were observed in PRN medicines management in terms of the type of medications prescribed and administered, residents’ underlying health conditions and needs, and the length of stay. However, differences in the studies’ methods and paucity of description of contextual factors affecting PRN processes make it difficult to compare findings and identify common patterns for PRN prescription and administration. Our review also showed variations in the type of psychotropic PRN medications prescribed. Such variation could be attributed to differences in medicines management routines, clinical reasoning, and personal judgments [1].

There were no reports as to the reasons underlying why PRN medications were prescribed and administered, but the absence of guidelines, and information as to the side effects and adverse drug reactions and “what to look out for” was notable. The use of unnecessary medications, excess dosing, and lack of monitoring in terms of medications’ effects and side effects have been highlighted as the most frequent medication-related problems, particularly for psychotropic medications [27,45]. Appropriate medication prescription for residents living in long-term care settings is a challenge for healthcare systems across the globe. It often increases the risk of adverse drug reactions (ADRs) and polypharmacy. Since many ADRs are preventable, some screening tools have been devised, including Beers’ Criteria and the Inappropriate Prescribing in the Elderly Tool (IPET), which augment professionals’ clinical judgement in medicine selection and prescription [46]. Profiling the adverse effects of psychotropic medications detects any changes in the patient following PRN administration [47,48]. Additionally, medicine reviews and checks for drug interactions, cautions, and doses have been suggested, including the Screening Tool of Older Persons’ Potentially Inappropriate Prescriptions (STOPP) [27,49], the Screening Tool to Alert to Right Treatment (START) [49,50] for older people, and the Norwegian General Practice (NORGEP) tool [51] for those under 71 years. However, PRN medicines management has not been incorporated into the above-mentioned monitoring tools. 

The interpretation of behavioural and other symptoms in residents and prediction of residents’ needs were mentioned as factors influencing how PRN medications were handled. However, in general, behaviours indicating the need for PRN medication administration were not clearly and specifically described. Additionally, it is unclear whether PRN administration is commenced by the nurse, the resident, or the family [52]. Prescription and administration of PRN psychotropic medications should not expose patients to the feelings of inferiority and coercion [3]. Since the decision-making process regarding PRN management is complex [11,12], there is a need for a decision support tool for PRN medicines management to prevent medication-related harm in long-term care settings: this should encompass systematic checks for the signs and symptoms of ADRs [47,48]. 

This review highlights the effect of a healthcare setting’s routines, and the education and training on how to handle PRN psychotropic medications’ administration and prescription. This is a general mandate for healthcare systems to devise and implement comprehensive national medicine policies to ensure safe and reliable medication prescription and administration [53], and to balance the risk and benefits of medications among residents [54]. However, there are often inconsistencies between prescription notes and administration records, which can overshadow attempts to assess the effectiveness of PRNs, due to a lack of data on the effect of PRNs on patients’ outcomes [52]. For example, there are different perceptions and opinions amongst physicians that prescribe, and nurses that administer, PRN medication [4,55]. Additionally, there are controversies regarding the use of PRN medications and non-pharmacological strategies including counselling, distraction, verbal and non-verbal de-escalation, massage, and education [4,5,29,56,57]. The improvement of PRN prescription practice and promotion of PRN prescription and administration requires changes in the healthcare policies of patient safety in care homes [27,58,59], including the mandate of formalised, systematic checking of residents for potential ADRs [47,48]. The implementation of system-level standards of practice for PRN administration, impact assessment, and documentation can help to improve overall patient safety in care homes [52]. Accountability for the administration of PRN medications can be improved by educating nurses in terms of vigilance over indications, reasons for use, medication effects and side effects, regular checking of prescribed medications, and continuous monitoring so as to avoid high dose medication and polypharmacy to ensure efficacy and prevent harm [9,60,61,62,63]. 

## 5. Conclusions

This systematic review focused on the PRN prescription and administration of psychotropic medications in long-term care settings. It used an integrative design, including both qualitative and quantitative research, to provide a more comprehensive overview of PRN and factors influencing it in practice. There is little information regarding the reasons underlying PRN deployment, and how related side effects and adverse reactions are identified and managed. Additionally, variations in the prevalence of PRN prescription and administration across long-term care settings indicate the absence of an appropriate and unified framework to be followed by all staff in the process of PRN medicines management. Accordingly, this review has identified a need to improve medicines management by exploring factors affecting PRN prescription and administration and by developing appropriate guidelines to prevent harm and to improve the quality and safety care delivered to those who live in long-term care facilities. This review has offered broader insights into healthcare provision, particularly nurses’ roles in monitoring patients and making decisions within collaborative environments of healthcare systems. This review has established the prevalence of PRN prescription and administration. Intervention studies, with qualitative components, are now needed to explore how healthcare providers and nurses can participate in daily care decisions about PRN medication prescription and administration and can monitor effects on residents’ health and wellbeing. 

## Figures and Tables

**Table 1:** Results of the systematic review.

Database	Total in Each Database	Title Selection	Abstract Selection	Full-Text Appraisal
PubMed (including Medline)	618	4	2	2
Scopus	105	29	5	5
Cochrane library	326	1	0	0
Cinahl	142	5	0	0
Norart (Nordic)	2	2	1	1
SweMed+ (Nordic)	3	2	0	0
IBECS (Spanish)	5	0	0	0
Cuiden (Spanish)	194	2	0	0
Medes (Spanish)	199	9	0	0
*Manual search/backtracking references*	0	0	0	0
Total	1594	54	8	8

**Table 2:** Papers selected for data analysis and synthesis.

Authors/Year	Country	Objective(s)	Method	Focus of Data Collection	Setting and Sample	Demographic Data
Snowdon et al. 2011 [37]	Australia	To understand and compare the patterns of psychotropic medications use in nursing homes.	A cross-sectional survey	Comparison of the patterns of psychotropic medication, using data from surveys from 1993, 1998, 2003, and 2009. Details of current prescription of medications during the last 14 days or since initiation of regular or PRN medication were recorded. Surveys reported the rate of PRN prescription and administration in the last 28 days. The administration of PRN medications at least 25 out of 28 days was considered regular medication use.	Number of nursing homes and percentage of their participation in the study were as follows: 46 (98%), 38 (97%), 51 (100%), and 44 (92%), in years 1993, 1998, 2003, and 2009, respectively.	Mean number of residents: 52.5, 52.0, 60.6, and 56.0, respectively. Percentages of 36% (*n* = 895, mean 78.7 years, SD = 12.1) and 64% (*n* = 1570, mean 84.2 years, SD = 9.6) were male and female, respectively. No information of their diagnosis was available.
Carder, 2012 [38]	USA	To identify if staff who were not registered nurses administered PRN medications to residents with dementia.	The qualitative analysis of interviews and medication record reviews	How is a decision on administering PRN medications to residents with dementia made? PRN administration staff were observed for 6 consecutive days, 16 h per day, including 72 scheduled medication rounds. Sixteen interviews were completed.	Sixteen unlicensed staff members supervised by registered nurses working at three assisted living settings with all 47 residents in urban and suburban areas.	Residents were mostly female. The mean age of staff was 38 years (SD = 8.3 years) and were mainly female (75%). Their job experience ranged 4 months to 8 years.
Rønningen et al., 2013 [39]	Norway	To document prescribing and administration of PRN medication in one nursing home, and to investigate how often, and how, the positive and negative effects of prescribed PRN medication are documented.	A cross-sectional survey of documentation of PRN medications	PRN prescription and administration prevalence. Description of documentation of PRN medication effects (beneficial and harmful) were documented in terms of quantity and quality.	PRN medicines management was documented and described for 108 patients in one nursing home over 15 weeks.	Mean age of residents was 84.5 years (SD = 9.4 years). They were mostly female (60.2%). No data on staff were provided.
Voyer et al., 2015 [40]	Canada	To identify behavioural and psychological symptoms in dementia that were associated with PRN antipsychotic medicine prescription and administration.	A secondary analysis on a prospective, observational, multisite cohort	Association between behavioural and psychological symptoms of dementia with PRN antipsychotic medicine use. Medication records of regular and PRN use of medication were reviewed. Atypical antipsychotic agents (risperidone (Risperdal®), olanzapine (Zyprexa®), and quetiapine (Seroquel®), and typical antipsychotic agents (haloperidol (Haldol®) were used. Prescription and administration of PRN antipsychotic agents during 7 days before the monthly assessment of behavioural and psychological symptoms of dementia assessments were considered.	A total of 146 nursing home residents from 7 settings. Subjects aged ≥65 years were included. Those without dementia, cognitive impairment, or behavioural and psychological symptoms were excluded.	Participants had a mean age of 85.6 years (SD = 7 years) and were mostly female (58.9%). They mainly were diagnosed with dementia (89.7%). Of 129 nurses, 76.7% were registered nurses and 90.7% were female. Their experience in geriatric wards was mainly greater than 10 years (72%).
Dörks et al., 2016 [41]	Germany	To examine the characteristics and potential predictors of PRN prescription and administration in nursing homes.	A multicentre survey	Investigation of characteristics and potential predictors of PRN medicine prescription and administration in nursing homes.	A total of 852 residents in 21 nursing homes organised by different institutions.	Their mean age was 83.5 years (SD = 10.5) and 76.5% were female. Their mean length of stay was 3.2 years (SD = 3.4).
Allers et al., 2017 [42]	Germany	To compare the use of antipsychotic medications in residents with and without dementia.	A cross-sectional survey	Assessment of the prevalence of antipsychotic medicine use and exploration of factors affecting their prescription. While data on all prescribed medications were collected from the residents’ medication schedules, antipsychotics prescribed as scheduled medication and on a PRN basis were studied and prescriptions of scheduled and PRN medication were compared.	All residents (*n* = 852) from 21 nursing homes without any exclusion criteria.	A percentage of 57.7% of the residents were diagnosed with dementia and their mean age was higher than those without it (84.9 vs. 81.4 years, but no standard deviations were presented). About three quarters of the residents were female and those with dementia were more commonly severely care-dependent (32.8 vs. 16.4%).
Westbury et al., 2018 [43]	Australia	To study the impact of an interdisciplinary intervention on the prescription of antipsychotics and benzodiazepines in older people’s residential care facilities.	A longitudinal study with comparisons over time	Investigation of the impact of an educational consultation intervention on the use of antipsychotics and benzodiazepines over 6 months. It assessed the possibility of substitution of medications prescribed regularly. The multi-strategic programme comprised: auditing psychotropic medication, staff education, and case reviews by the physician, pharmacist, and nurse at the beginning of the programme and at 3 and 6 months.	A national-level sample consisting of 150 older people’s residential care facilities hosting 12,157 people.	The residents’ mean age was 85.8 years (SD = 8.6).
Westbury et al., 2019 [44]	Australia	To analyse the use of psychotropics in a national sample of residential aged care facilities.	A retrospective cohort	Analysis of psychotropic use in a large national sample of residential aged care facility residents, derived from a project to promote the appropriate use of antipsychotics and benzodiazepines. A multi-strategic interdisciplinary intervention was devised consisting of a 6-month programme with cycles of audits, education, and a review of sedatives. Prescribing data were extracted via a custom-made website.	A large national-level sample of 150 residential aged care facilities consisting of 11,368 residents	Data on psychotropic prescribing was collected from for 139 of 150 facilities with a response rate of 93%. Clinical, diagnostic, and demographic data were not reported.

PRN: *pro re nata*; SD: standard deviation.

**Table 3:** Prevalence and type of PRN prescription and administration.

Authors/Year	Prevalence of PRN Prescription	Prevalence of PRN Administration	Medicines Prescribed or Administered PRN
Snowdon et al., 2011 [37]	Mean number of 1.3 per resident Mean number of 0.9 when topical applications such as ear, eye, nose, and dermatological preparations were excluded (no measures of dispersion reported.)	Once or more in every 5 prescriptions	Clonazepam and midazolam for six residents and antipsychotic and/or anxiolytic medications including haloperidol to 3.3% of residents and Risperidone to 1%. Numerators and denominators were not reported.
Carder, 2012 [38]	Nearly all residents had a PRN order, with a range from 0 to 14 per resident, and with a mean of 5.82 (no measures of dispersion reported.)	No data	No data
Rønningen et al., 2013 [39]	1.1% (*n* = 183) of prescriptions	519 of the 839 (61.9%)	Psychotropic medications such as oxazepam, clomethiazole, diazepam, zopiclone, tramadol, morphine, and oxycodone were among the most frequently prescribed and administered medications as different brand names or pharmaceutical formulations (e.g., tablets and suppositories). Percentages, numerators, and denominators were not reported.
Voyer et al., 2015 [40]	19.9% of prescriptions	No data	Antipsychotics including risperidone (37.9%), haloperidol (34.5%), quetiapine (10.3%), olanzapine (10.3%), haloperidol, and quetiapine (3.4%) were prescribed. After 5 months, olanzapine was replaced by risperidone (3.4%). Numerators and denominators were not reported.
Dörks et al., 2016 [41]	A total of 2117 (27.9 %) prescriptions were PRN. Additionally, 638 (74.9 %) received at least one PRN medication. Each resident was treated with a mean of 2.5 ± 2.3 PRN medications.	No data	Lorazepam was prescribed to 67 (7.9%) of residents with a mean duration of 579 ± 627 days.
Allers et al., 2017 [42]	A percentage of 35.9% of prescriptions to residents with dementia vs. 23.0% for those without dementia were PRN.	No data	23.8% of residents with dementia and 5.7% without dementia were prescribed PRN antipsychotics. Only a small percentage of residents without dementia received PRN antipsychotics alone, without any scheduled antipsychotic medications (3.1%), a lower proportion than residents with dementia (10.8%). Residents with dementia were prescribed both scheduled and PRN antipsychotics more often than residents without dementia (13.0 vs. 2.5%). Of typical antipsychotics, melperone and promethazine were most often prescribed. Numerators and denominators were not reported.
Westbury et al., 2018 [43]	PRN antipsychotics were prescribed to 10.8% (9.5-12.1%) and benzodiazepines to 30.1% (27.6-32.6%) of residents.	No data	Antipsychotics, excluding lithium and prochlorperazine, and all types of benzodiazepines were converted to diazepam equivalents, but no separate data on each medication were provided.
Westbury et al., 2019 [44]	Of 11368 residents, 1261 (11.1%) and 3461 (30.5%) were prescribed PRN antipsychotics and PRN benzodiazepines, respectively.	No data	A percentage of 11.1% of residents were prescribed PRN atypical antipsychotics, including risperidone, quetiapine, and olanzapine, and typical antipsychotics such as haloperidol. Benzodiazepine, as an anxiolytic, prescribing included oxazepam, diazepam, and alprazolam to 17.9% of residents. Hypnotics, temazepam, and nitrazepam were prescribed to 16.4%. Both regular and PRN benzodiazepines were prescribed to 1150 residents (47%). Additionally, 724 (29%) of them with regular antipsychotic orders were prescribed extra doses of PRN.

PRN: *pro re nata*.
